# Ectopic Adrenal Cortical Tissue in the Ovary: A Rare Incidental Histopathologic Finding

**DOI:** 10.3390/diagnostics16203275

**Published:** 2026-10-09

**Authors:** Dae Hyun Song, In Ae Cho, Jong Chul Baek

**Affiliations:** 1 Department of Pathology, Institute of Health Science, School of Medicine, Gyeongsang National University, Jinju 52727, Republic of Korea; golgy@hanmail.net; 2 Department of Pathology, Gyeongsang National University Changwon Hospital, Changwon 51472, Republic of Korea; 3 Department of Obstetrics and Gynecology, Institute of Health Science, School of Medicine, Gyeongsang National University, Jinju 52727, Republic of Korea; obgychoia@gnu.ac.kr; 4 Department of Obstetrics and Gynecology, Gyeongsang National University Hospital, Jinju 52727, Republic of Korea; 5 Department of Obstetrics and Gynecology, Gyeongsang National University Changwon Hospital, Changwon 51472, Republic of Korea

**Keywords:** ectopic adrenal cortical tissue, ovarian adrenal cortical rest, ovary, Melan-A, inhibin-α

## Abstract

Ectopic adrenal cortical tissue (EACT) is an uncommon developmental heterotopia and is exceptionally rare in the ovary of adult women. A 52-year-old woman, 6 years after menopause, was evaluated for progressive enlargement of a uterine leiomyoma and postmenopausal spotting. Transvaginal ultrasonography and pelvic magnetic resonance imaging demonstrated multiple uterine leiomyomas but no adnexal mass or endometrioma. During total laparoscopic hysterectomy with bilateral salpingo-oophorectomy, dense adhesions between the sigmoid colon and posterior cul-de-sac were unexpectedly encountered. Histopathologic examination of the adhesion-related tissue revealed neither endometriosis nor significant inflammatory change. Microscopic examination of the ovary identified a 1.9 mm, sharply circumscribed nodule composed of clear, vacuolated zona fasciculata-like cells and compact eosinophilic zona reticularis-like cells. The lesional cells showed diffuse Melan-A immunoreactivity and focal-to-patchy inhibin-α positivity, supporting adrenocortical differentiation. The findings established a diagnosis of ovarian EACT. This case emphasizes that microscopic ovarian adrenal rests may be radiologically occult and should be distinguished from ovarian steroidogenic neoplasms by integrating clinical, morphologic, and immunohistochemical findings.

**Figure 1:**
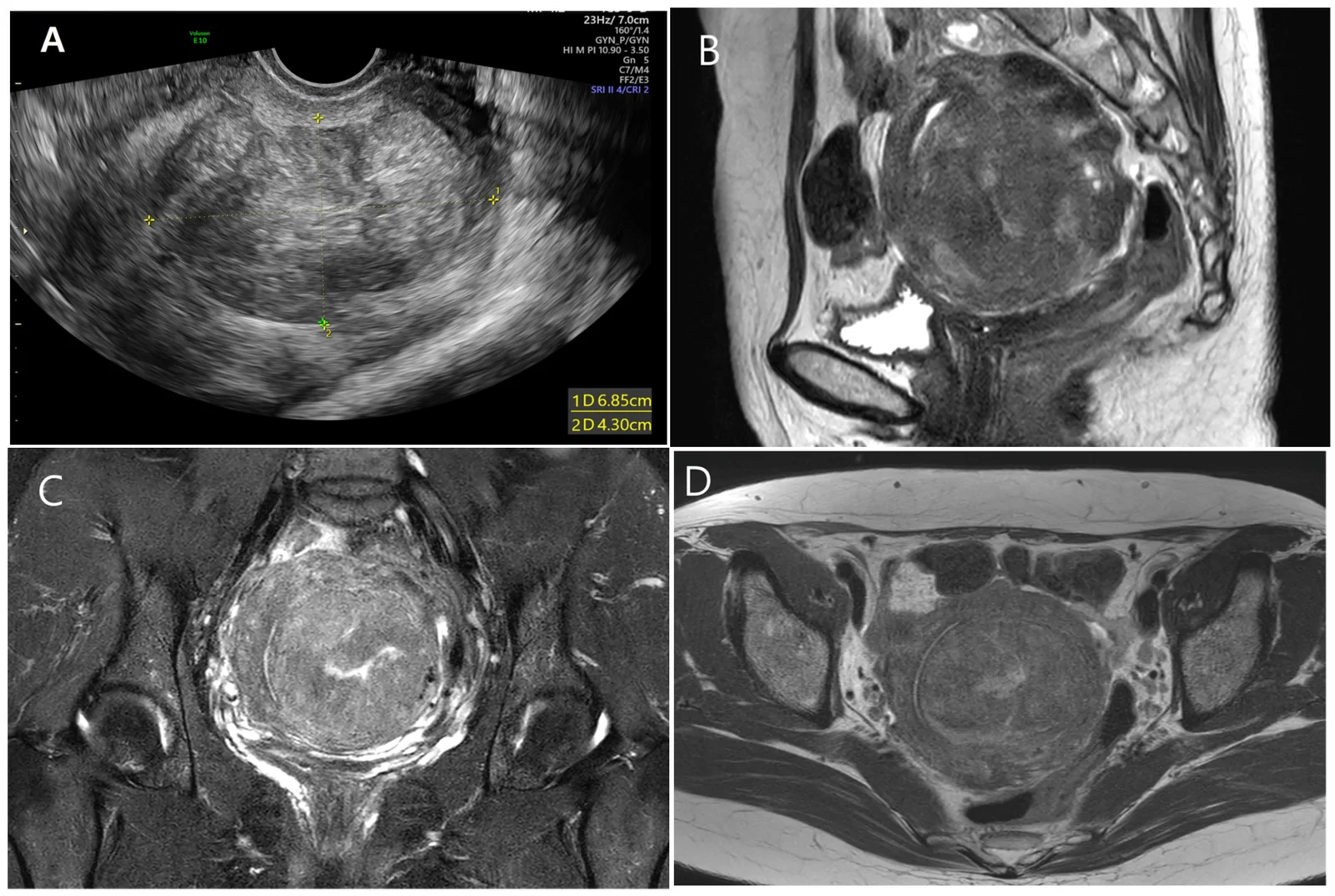
Preoperative ultrasonographic and magnetic resonance imaging findings. A 52-year-old woman, 6 years after menopause, was evaluated for a uterine leiomyoma that had enlarged from approximately 4.3 cm to more than 6 cm after menopause and was accompanied by recent postmenopausal spotting. She had two prior vaginal deliveries and no history of abdominal or pelvic surgery, endocrine disease, or symptoms of adrenal hormone excess. (**A**) Transvaginal ultrasonography showing a 6.85 cm uterine leiomyoma and an endometrial thickness of 5 mm, without a definite adnexal mass. (**B**) Sagittal T2-weighted magnetic resonance image showing the dominant well-circumscribed uterine leiomyoma. (**C**) Coronal T2-weighted image demonstrating the dominant lesion with additional myometrial nodules. (**D**) Axial T2-weighted image showing the enlarged myomatous uterus without an adnexal lesion. No adnexal mass, endometrioma, or other significant pelvic abnormality was identified. Endometrial biopsy revealed no significant pathologic abnormality. Because of continued postmenopausal enlargement of the uterine mass and associated spotting, surgical treatment was undertaken.

**Figure 2:**
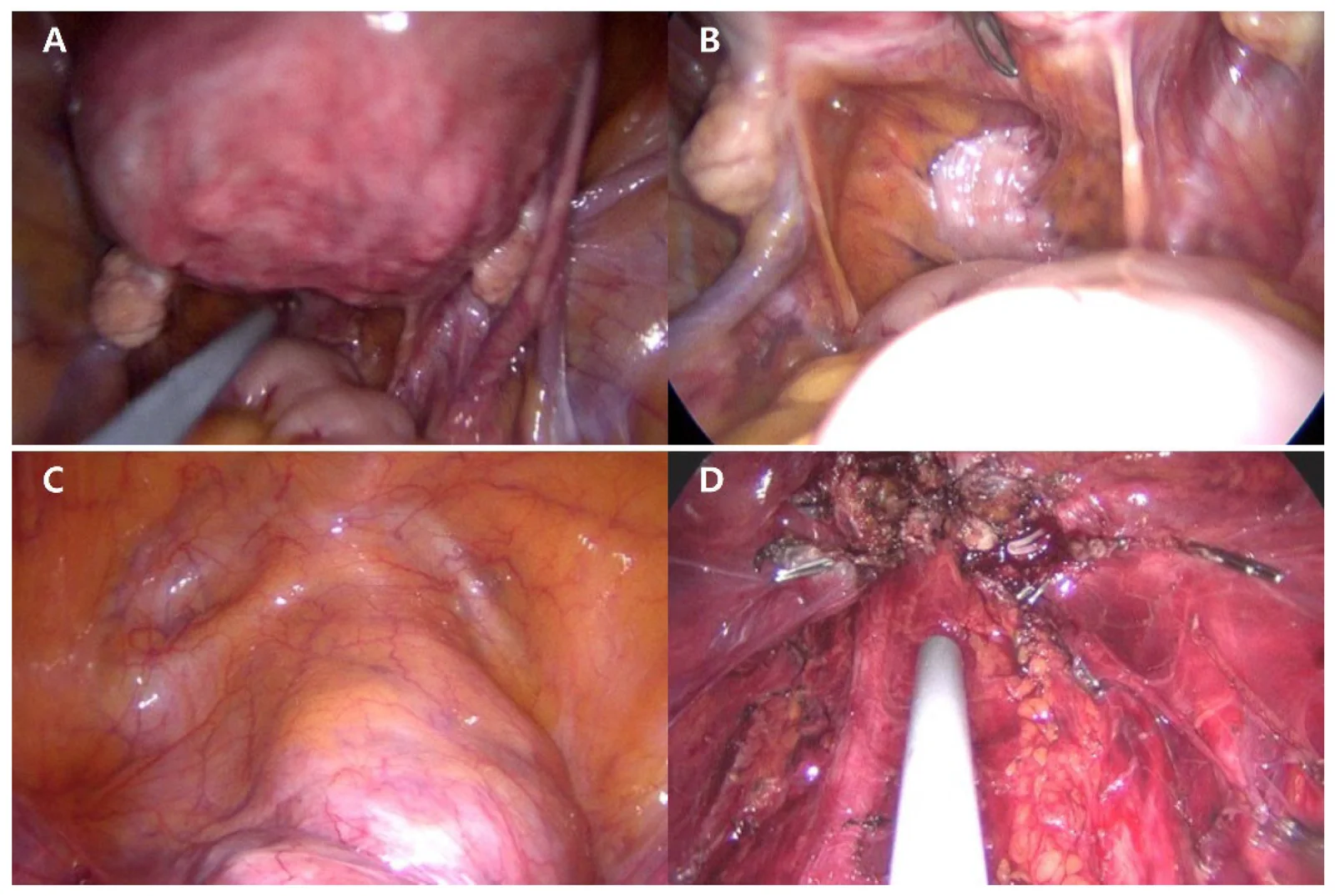
Intraoperative laparoscopic findings. The patient underwent total laparoscopic hysterectomy with bilateral salpingo-oophorectomy. Laparoscopy demonstrated a myomatous uterus and a grossly unremarkable ovary without periovarian adhesions (**A**). Dense adhesions tethered the sigmoid colon to the posterior cul-de-sac and obscured the usual anatomy of the uterosacral ligaments (**B**), whereas the anterior cul-de-sac and urinary bladder were free of significant adhesions (**C**). After hysterectomy, complete adhesiolysis was achieved without sigmoid colon injury (**D**), and tissue from the adhesion sites was submitted for histopathologic examination.

**Figure 3:**
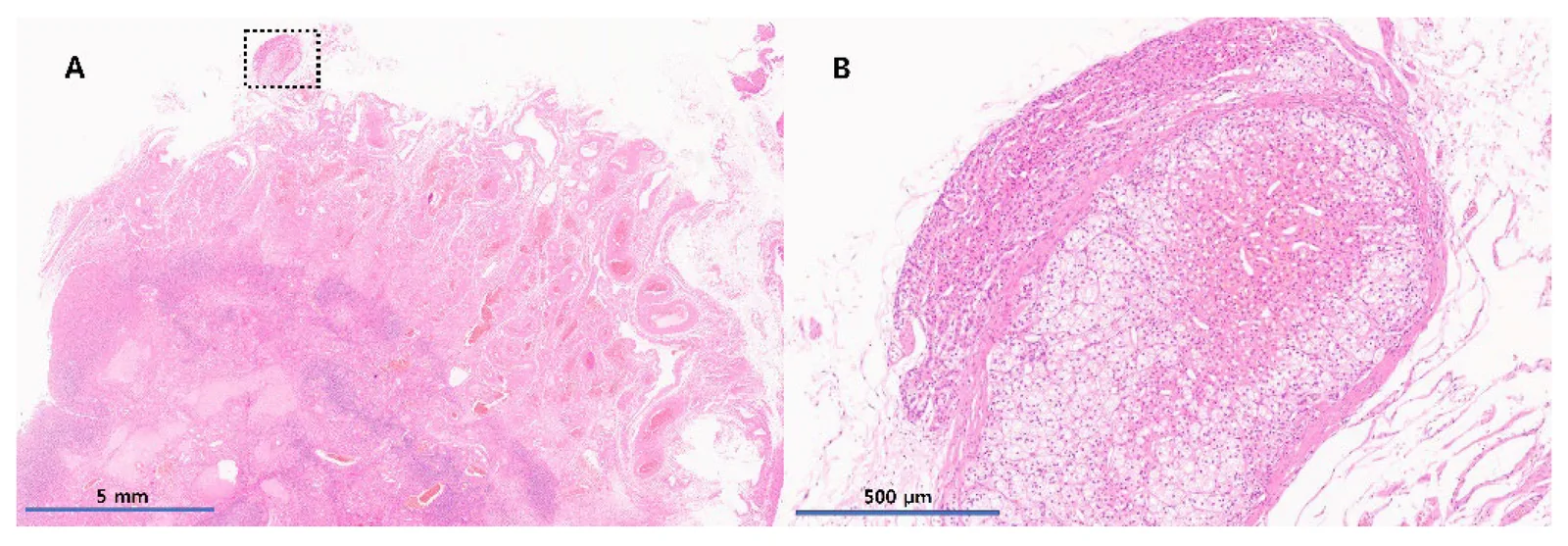
Histopathologic features of ectopic adrenal cortical tissue in the ovary. (**A**) Low-power view showing a 1.9 mm, well-circumscribed nodule at the ovarian periphery (dotted rectangle) (H&E, ×1; scale bar, 5 mm). (**B**) Higher-power view showing a sharply demarcated lesion composed of clear to finely vacuolated polygonal cells and smaller eosinophilic granular cells, resembling the zona fasciculata and zona reticularis, respectively. No adrenal medullary component is identified (H&E, ×50; scale bar, 500 μm).

**Figure 4:**
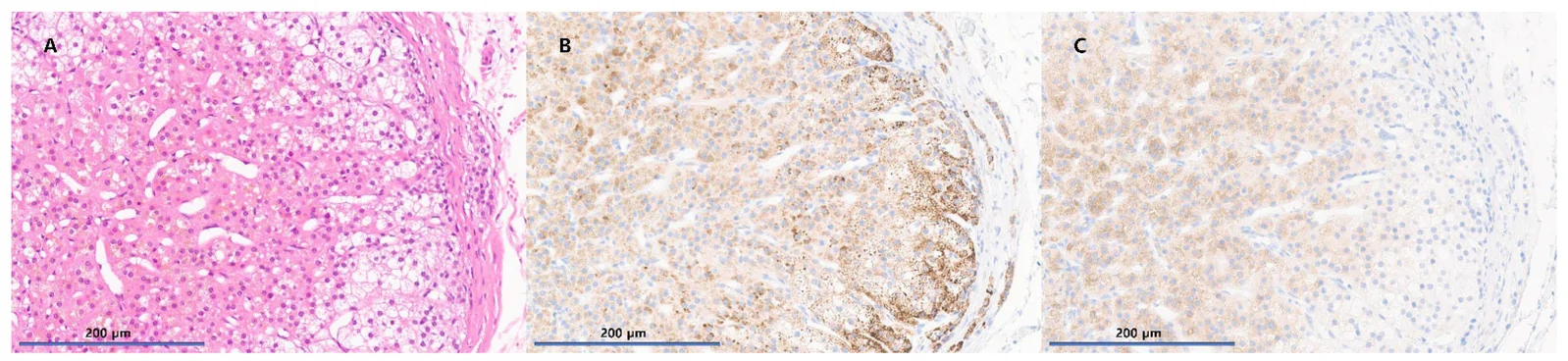
Histologic and immunohistochemical features of ectopic adrenal cortical tissue. (**A**) High-power H&E image showing large clear-to-vacuolated polygonal cells admixed with smaller eosinophilic cells, with focal lipofuscin-like pigment and no significant atypia or increased mitotic activity (×200; scale bar, 200 μm). (**B**) Diffuse cytoplasmic Melan-A immunoreactivity in the lesional cells (×200). (**C**) Focal-to-patchy cytoplasmic inhibin-α immunoreactivity supporting adrenocortical differentiation (×200). Because of the unexpectedly extensive pelvic adhesions, tissue obtained from the adhesion sites was examined thoroughly; no endometriosis, endometrioma, or significant inflammatory disease was identified. The contralateral ovary was grossly and histologically unremarkable. No significant clinical abnormalities or clinical manifestations of adrenal hormone excess were noted during postoperative follow-up of less than 1 year. The combined morphologic and immunophenotypic findings supported a diagnosis of ectopic adrenal cortical tissue of the ovary. Ectopic adrenal cortical tissue is a developmental heterotopia believed to result from abnormal separation or migration of adrenal cortical cells during embryogenesis. Because the adrenal cortex and gonads arise in close proximity within the adrenogonadal primordium, displaced cortical tissue may persist along the developing urogenital tract [1,2]. Ectopic adrenal tissue has also been described at more distant sites, including the lung, mediastinum, gastric wall, liver, placenta, and spinal canal [2,3]. Most ectopic rests consist exclusively of cortical tissue, consistent with separation before neural crest-derived medullary cells enter the developing adrenal gland [2,4,5]. In the female genital tract, adrenal rests have been reported in the ovary, broad ligament, paraovarian region, and fallopian tube; ovarian involvement in adults remains particularly uncommon and is usually discovered incidentally during surgery or histopathologic examination [6,7,8,9]. Incidental ectopic adrenal rests are generally benign and clinically nonfunctioning, their clinical significance depends on lesion size, location, and endocrine activity [2,3,4]. The present lesion should be distinguished from an ovarian adrenal rest tumor (OART), which has been described predominantly in patients with congenital adrenal hyperplasia and chronic adrenocorticotropic hormone stimulation. OARTs may be bilateral or hormonally active and may present with persistent virilization even when pelvic imaging is unrevealing [10,11]. Because OARTs can closely resemble Leydig cell tumors, stromal luteomas, and steroid cell tumors, accurate diagnosis requires careful integration of the clinical setting with histopathologic and immunohistochemical findings [11]. Larger OARTs, particularly those associated with congenital adrenal hyperplasia, may be visible on MRI as T1-isointense and T2-hypointense masses with early enhancement followed by rapid washout [12]. Conversely, microscopic ectopic adrenal cortical tissue may remain below the detection threshold of ultrasonography and MRI [10]. In the present case, no ovarian lesion was identified preoperatively, whereas histologic examination subsequently revealed a 1.9 mm focus of EACT. The absence of endocrine manifestations, the microscopic size, and the lack of an imaging correlate favor incidental EACT rather than a clinically significant OART. The lesion was clinically nonfunctioning; however, biochemical steroidogenic activity was not specifically assessed. Histologically, EACT is characterized by a circumscribed population of bland steroidogenic cells that recapitulates adrenal cortical differentiation. Larger polygonal cells with lipid-rich, clear to finely vacuolated cytoplasm resemble zona fasciculata cells, whereas smaller compact eosinophilic cells resemble the zona reticularis; an adrenal medullary component is often absent [4,6,7,8]. When grossly apparent, these lesions are typically small, circumscribed, and yellow to yellow-brown because of the lipid-rich cortical cytoplasm [6,9]. Ovarian EACT has occasionally been reported in association with unrelated epithelial ovarian tumors, including serous cystadenoma and borderline mucinous cystadenoma [13,14], indicating that an adrenal rest may coexist with another ovarian lesion without constituting part of the primary neoplasm. Immunohistochemistry is supportive but should not be interpreted in isolation. Ectopic adrenal cortical cells commonly express Melan-A, inhibin, calretinin, and steroidogenic factor-1. In this case, diffuse Melan-A staining and focal-to-patchy inhibin-α expression, together with fasciculata-like and reticularis-like morphology, supported adrenocortical differentiation [7,15]. Because Melan-A and inhibin may also be expressed in ovarian steroidogenic and sex cord-stromal tumors, marker positivity alone is insufficient for diagnosis [15,16]. SF-1, a more specific marker of adrenocortical differentiation, was not performed because the antibody was not available at our institution and the amount of residual tissue was limited. This represents a diagnostic limitation of the present case. The differential diagnosis includes steroid cell tumor, Leydig cell tumor, and stromal luteoma. Steroid cell tumors typically show sheets or nests of steroidogenic cells without characteristic adrenal cortical zonation, whereas Leydig cell tumors may contain Reinke crystals and are usually composed of eosinophilic polygonal cells. Stromal luteomas arise within the ovarian stroma and are composed of luteinized stromal cells. In contrast, EACT is characterized by well-circumscribed fasciculata-like and reticularis-like zonation, which, together with its minute size and bland cytology, supports the diagnosis. Because ovarian steroidogenic tumors may also express Melan-A, inhibin, and calretinin, immunohistochemical findings should be interpreted together with the characteristic adrenal cortex-like morphology and clinical context [15,16,17]. An additional intraoperative finding was dense adhesion between the sigmoid colon and posterior cul-de-sac despite the absence of previous pelvic surgery or a history of pelvic inflammatory disease. The dense pelvic adhesions prompted histopathologic evaluation to exclude endometriosis or inflammatory disease; however, no specific pathologic abnormality was identified. Therefore, the adhesions were considered an incidental operative finding unrelated to the ovarian EACT. Given that EACT is believed to result from aberrant separation or migration of adrenocortical precursor cells during embryogenesis, there is no established developmental mechanism linking EACT to pelvic adhesion formation [2]. The adhesions were therefore considered an independent incidental finding of undetermined etiology. Previously reported cases of ovarian ectopic adrenal tissue have occurred across a wide age range and in diverse clinical settings (Appendix A Table A1). Several were incidentally associated with ovarian neoplasms, whereas others were identified in otherwise unremarkable ovaries. In the present case, the 1.9 mm lesion was neither radiologically nor grossly detectable and was identified only on microscopic examination. These findings emphasize that accurate diagnosis of microscopic ovarian EACT requires integration of the clinical context, characteristic adrenal cortical morphology, and immunohistochemical findings.

## Data Availability

The data presented in this study are available on request from the corresponding author. The data are restricted to protect patient privacy and confidentiality, as this study is a case report involving individual patient information.

## Appendix A

**Table A1:** Comparison of the present case with previously reported cases of ovarian ectopic adrenal tissue.

Reference	Age	Clinical Presentation/Associated Lesion	Size
Gharib et al., 2025 [6]	54 y	Endometrial cancer; incidental ovarian lesion	6 mm
Lee et al., 2021 [7]	68 y	Pelvic organ prolapse; incidental ovarian lesion	3 × 2 mm
Chew et al., 2017 [14]	22 y	Abdominal distension; borderline mucinous cystadenoma	Not reported
Şahin et al., 2017 [13]	65 y	Pelvic pain/postmenopausal bleeding; bilateral serous cystadenomas	Not reported
Yasar et al., 2012 [18]	50 y	Menorrhagia, dyspareunia, Granulosa cell tumor of contralateral ovary	Not reported
Usta et al., 2006 [19]	21 y	pelvic pain and menstrual irregularity.; 25 cm serous cystadenoma	2 × 1 mm
Present case, 2026	52 y	Postmenopausal spotting; uterine leiomyoma; incidental ovarian lesion	1.9 mm

Recognition of ovarian EACT is clinically important because this benign developmental rest may be radiologically occult and can histologically mimic steroidogenic or clear-cell ovarian neoplasms. Accurate diagnosis requires integration of the clinical context, operative findings, characteristic adrenal cortex-like morphology, cytologic features, and immunohistochemical profile rather than reliance on a single marker. Correct identification can prevent overdiagnosis and unnecessary oncologic evaluation or treatment while facilitating independent assessment of unrelated pelvic abnormalities.
